# Novel approach on reduction in GHG emissions from sludge lime stabilization as an emergent and regional treatment in China

**DOI:** 10.1038/s41598-018-35052-9

**Published:** 2018-11-08

**Authors:** Hongtao Liu

**Affiliations:** Institute of Geographic Sciences and Natural Resources Research, Chinese Academy of Sciences, Beijing, 100101 China

## Abstract

As a typical organic solid waste, sludge plays an important role in contributing to greenhouse gas (GHG) emissions resulted by its treatment and disposal. As a temporary and emergent treatment measurement, sludge lime stabilization is regionally adopted in most sludge generated units in China. In present case, sludge lime stabilization system in China was productive of total GHG emissions, including indirect and direct emissions during lime stabilization and carbon reduction owing to lime synthesis and consumption, were first quantified respectively. The results indicated that electricity consumption was main component of indirect emissions, including mixing and transportation related mechanical equipment use. Direct emission was attributed to CO_2_ absorption during the second step in hydration reaction of lime stabilization. Meanwhile, a carbon credit portion of lime synthesis was also taken to the consideration of carbon budget. In brief, reduction in total replaceable carbon emission resulted by sludge lime treatment in comparison to landfill was calculated to be 0.8092 tCO_2_e·t^−1^. As treated production, lime-stabilized sludge is suggested to amend acidic soil for its revegetation. It is concluded that lime stabilization of sludge shows a significant GHG reduction effect despite of its temporary and emergent nature.

## Introduction

It is often faced that high environmental risk and the lack of treatment facilities when more and more sludge is generated along with a large amounts of sewage daily, especially in some developing countries, such as China. By September 2016, the capacity of sewage treatment of China had reached 170 million cubic meters per day^1^; therefore, the corresponding sludge production had accumulated up to 100 thousand tons per day. Thus, a remarkable figure of 36 million 500 thousand tons every year can be cited in the elaboration for annual sludge production in China. It is not realistic that such huge sludge can be timely treated by harmless measures and in recycling disposal ways. Under this background, some temporary, emergent and effective techniques arose. For instance, lime stabilization is one of acceptable and regionally adopted in sludge treatment sites especially in north China. Sludge lime stabilization is of some advantages including significant drop in sludge water content and volume^2^, good sterilization^3,4^. In some extent, the effects of contaminants in sludge can be also attenuated when lime was added into sludge^5^. As a result, possible environmental risk is correspondingly weakened if the lime added sludge was reused as amendment to soil. Especially, in the inactivation of heavy metal, the lime played an important role in its bio-availability regulation^6^, even it was called ‘heavy metal deactivator’^7^. In addition, when lime treated sludge was amended in acid soil, the reduction on heavy metal mobility in soil^8^ and the elevation in growth and yield were also anticipated^9,10^. In other word, lime was introduced to the improvement on biomass of plant grown on lime-stabilized sludge amended soil and the heavy metal toxicity attenuation in some designated cases.

However, lime stabilization remains regarded as a transitional and impermanent measure as far as sludge disposal is concerned. Subsequent difficulty should be faced again that how to dispose lime stabilized sludge reasonably and how to choose its options among incineration, bio-treatment (such as aerobic compost) and landfill. The outlet of lime stabilized sludge seems to be some problems^2,11^, for instance, incremental results shown that if sludge incineration is accepted solution, but lime addition prior to incineration is unnecessary^12^. All the same, sludge lime treatment is widely used, particularly in developing countries. It is attributed to the facility absence of sludge treatment and recycling or the shortage of fund for the sludge treatments.

If this has happened, the GHG emissions of sludge lime stabilization should be had the initiative in our hands. But the blank was found regretfully in previous reports and documents regarding sludge lime stabilization even lime treated organic waste. It is an important omit for total GHG emissions from the system of sludge treatment and disposal. Unfortunately, so far, the standard method of GHG calculation of sludge lime stabilization has not been taken to the consideration of IPCC official documents. The objective of this work is to make a quantization of GHG from sludge lime stabilization according to statistical data obtained in practical project and previous related research results. Meanwhile, it is compared with GHG from baseline scenario of native sludge disposal to obtain the net GHG emissions of lime stabilization technique, proposing the position and the priority order of lime stabilization in the chain of GHG emissions sludge management.

## Results

### Indirect GHG emissions from sludge landfill process

In China, the water content of sludge is required to reduce below 60%, being qualified for landfill disposal, therefore indirect GHG emissions from electricity consumption on-site (PE_*elec,y*_) in present case was mainly attributed to sludge dewatering. In term of formula (2), PE_*elec,y*_ was calculated to 0.0068 tCO_2_e∙t^−1^. The GHG emissions due to fossil fuel burning on-site (PE_*fuel,y*_) were mainly brought by machine activities related landfill such as excavators, bulldozers, compactors and rotavators. According to the formula (3) appeared in 2.1, PE_*fuel,y*_ was quantified to 0.0238 tCO_2_e∙t^−1^. Leakage in sludge transportation is another source of indirect emission. In this case, the distance was assumed to be 20 kilometers between the sewage plant and the site of sludge landfill, and it was defaulted to 5 t for loading per sludge truck. Based on above conditions and combined with formula (4), PE_*tran,y*_ was calculated to 0.0028 tCO_2_e∙t^−1^. In sum, total indirect GHG emissions from sludge landfill were determined to 0.0334 tCO_2_e∙t^−1^ (see Table 1).

**Table 1:** Make-up of indirect GHG emissions from sludge landfill.

Component unit	Quantity of GHG emissions (tCO_2_e∙t^−1^)
Electricity consumption (PE_*elec,y*_)	0.0068
Fossil fuel consumption (PE_*fuel,y*_)	0.0238
Transportation leakage (PE_*tran,y*_)	0.0028
Total	0.0334

### Direct GHG emissions from sludge landfill process

In general, anaerobic degradation occurred in sludge landfill process during which considerable amount of methane and CO_2_ were produced. These CO_2_ emissions are not taken to the consideration of national totals, because this part of carbon is generated from biogenic cycle and net emissions can be explained according to the Agriculture, Forestry and Other Land Use Sector. The methane collection and utilization systems were not installed at landfill site in this case, so the acquiesce value of MD_reg,y_ was defined to 0, and the value of “f” in the formula of MB_y_ was also defaulted to 0. Moreover, based on the climatic feature of Qian’an city, an annual average temperature of below 20 °C reported by IPCC and a proportion of annual precipitation as well as potential evapo-transpiration (<1), the value of “K” in the formula to determine MB_y_ was assigned to 0.06. Overall, on basis of the formulas (5) and (6), the direct emission intensity due to sludge landfill was finally calculated to 0.6776 tCO_2_e∙t^−1^.

### Indirect GHG emissions related to sludge lime treatment

**Figure 1:**
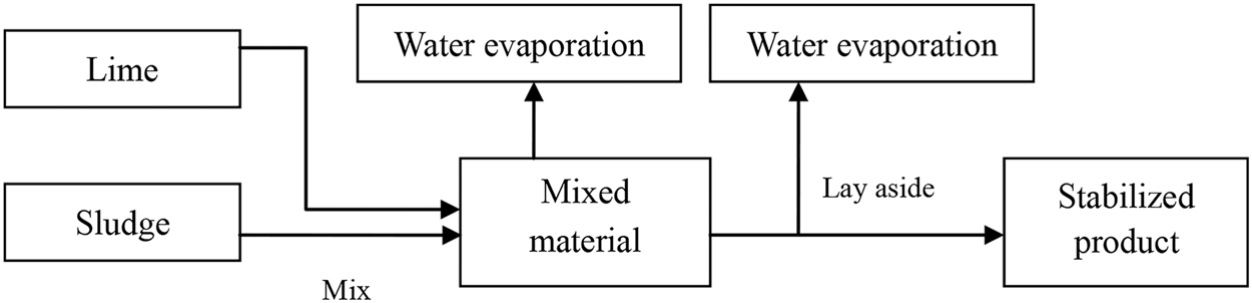
Process flow chart of sludge lime stabilization.

Mechanical operation related lime treatment resulted in electricity consumption, which are mainly attributed to indirect GHG emissions, such as sludge and lime mixing, transport and loading as well as gauge system. Despite auto-control technology was widely employed in most of lime stabilization, many mechanical and electrical activities are still necessary to finish lime stabilization process (Table 2). Therefore, PE_*elec,y*_ was quantified as 0.0161 tCO_2_e∙t^−1^. The amount of diesel oil consumed was low because there were no dump trucks or forklifts in this process; thus, the value of PE_*fuel,y*_ was determined to be 0.0004 tCO_2_e∙t^−1^. As process flow shown in Fig. 1, lime stabilized sludge was developed and the residue part evaporated mainly as from of water. In addition, the distance between the sewage plant and sludge treatment site was assumed to be 20 and that between the treatment site and lay site was assumed to 10 kilometers. Accordingly, the leakage value in sludge transportation (PE_*tran,y*_) was calculated to be 0.0039 tCO_2_e∙t^−1^. Overall, this portion of the GHG emissions was determined to 0.0204 tCO_2_e∙t^−1^ (As shown in Table 3).

**Table 2:** Electricity consumption by machines and electronic equipment associated with lime stabilization process.

Functional unit	Equipment or instrument	Electricity consumption statistic (KWh∙t^−1^)
Material mixing	Mixer	3.85
Sludge transport feeding	Belt conveyor	0.20
	Bunker	
Lime transport feeding	Belt conveyor	0.15
	Bunker	
operational control system	Temperature/water related monitoring; Electric valve; control computer	0.02
Mixture transport outlet	Belt conveyor	0.18
	Bunker	
Gauge system	flow counter	0.01
Total		4.41

**Table 3:** Make-up of indirect GHG emissions from sludge lime treatment.

Component unit	Quantity of GHG emissions (tCO_2_e∙t^−1^)
Electricity consumption (PE_*elec,y*_)	0.0161
Fossil fuel consumption (PE_*fuel,y*_)	0.0004
Transportation leakage (PE_*tran,y*_)	0.0039
Total	0.0204

### Direct GHG emissions from sludge lime stabilization process

**Figure 2:**
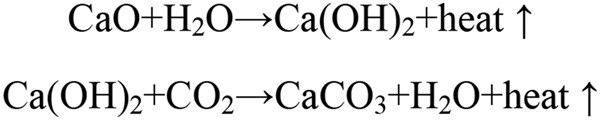
Hydration reaction chemical equation of lime stabilization.

Theoretically, the GHG from sludge lime stabilization primarily consists of the emissions of CH_4_ and N_2_O. However, the process is hydration reaction (Fig. 2). Accordingly, only one equivalent CO_2_ (compared with CaO) was consumed in the second step of sludge lime stabilization process. Therefore, no CH_4_ and N_2_O generations are considered in term of the lime stabilization chemical equation (Fig. 2). And the value of PE_*c,CH4,y*_ and PE_*c,N2O,y*_ were all assigned to 0. Consequently, remaining negative loss of CO_2_ represented the direct emissions from this process. As a result, PE_*c,y*_ from the sludge lime stabilization was calculated to be −0.0786 tCO_2_e∙t^−1^.

Beside this portion of GHG, there is another portion of material (lime) consumption in the lime stabilization i.e. PE_*lime,y*_, which should also be taken to the consideration. As shown in Table 4, the optimal addition proportion of lime to sludge was compared and screened, and 10% was obtained as threshold value for significant water content reduction and pH elevation as well as no Fecal coliform bacteria exiting. According to formula (9) and lime addition proportion of 10%, the value of lime synthesis and consumption resulted carbon capture was quantified to be 0.04 tCO_2_e∙t^−1^.

**Table 4:** Effect of different addition proportion of lime on the sludge stability.

Lime addition proportion	Fecal coliform bacteria (MPN·100 g^−1^)	Water content	pH	Sense of odor
5%	nd*	61.8%	12.1	Easily smell slight odor
10%	nd	57.3%	12.5	Narrowly smell slight odor
15%	nd	56.2%	12.6	Narrowly smell slight odor
20%	nd	54.6%	12.8	Narrowly smell slight odor

*nd: no detection.

### Total carbon budget from sludge lime stabilization in comparison to landfill

In this case study, direct and indirect emissions were made of the carbon debit, but the carbon credit only consisted of the CO_2_ absorbed in the process of lime stabilization. Total emissions caused by landfill were quantified to be 0.711 tCO_2_e∙t^−1^, while those from lime stabilization were −0.0982 tCO_2_e∙t^−1^ (as shown in Table 5). These findings are attributed to CO_2_ uptake happened in the second step of chemical reaction process of lime stabilization. Consequently, a decrease of 0.8092 tCO_2_e∙t^−1^ sludge was obtained by sludge lime treatment instead of landfill disposal.

**Table 5:** Comparison of carbon budget between lime stabilization and baseline scenario (landfill).

Sludge treatment or disposal option	Carbon debit (tCO_2_e∙t^−1^)		Carbon credit (tCO_2_e∙t^−1^)	Total emission (tCO_2_e∙t^−1^)
	Indirect emission	Direct emission		
Baseline scenario (Landfill)	0.0334	0.6776	—	0.711
Lime stabilization	0.0204	−0.0786	0.04	−0.0982
Total emission reduction				0.8092

## Discussion

Lime treatment is an effective technique to achieve the volume reduction of sludge. It holds the advantages featuring most cost-effective, easily practical operation with high dryness fraction and good stability^2,13^. However, sludge lime stabilization should be considered objectively, the characteristics of sludge lime treatment also should be divided into two parts. Beside the merits mentioned above, there are still some drawbacks, such as the outlet of lime treated sludge. In practice, it usually increases the pH value and leads to soil compaction after soil treatment after it was applied to soil. In some extent, it is unfriendly to the soil environment and unbeneficial to plant growth. All the same, lime treatment of sludge was employed as an improvised makeshift and even popularly in past decades because of overdue construction of sludge treatment plants and the absence of more suitable technical route in many developing countries. The same is true of China; particular in recent ten years, more and more a huge of sludge was generated. Some part of those have to be treated by provisional measure, such as lime stabilization so that discard in random, in some extent, can be avoid, implying that more serious environment pollution by being thrown it aside can be attenuated. So far, lime stabilization remains ubiquitous at considerable proportion in northern region of China. However, the evaluation and investigation on GHG emissions are rarely to be found in referenced documents. It is only one report solely said that sludge composting with a few lime as consortium of additives were found to emit very less amount of GHG and gave the highest maturity than other treatments^14^. Thus it is adequately and clearly shown that the GHG emissions resulted from lime stabilization was not paid enough attention.

In previous report, GHG emissions of sludge composting also did not include CH_4_ and N_2_O, meanwhile, there was generated CO_2_ during organic matter decomposing process in composting, however this part of the emissions was attributable to the short-term carbon cycle, implying that no elevation of atmospheric CO_2_ happened due to composting, and it was not taken to consideration of carbon emission budgets system^15–17^. Significantly differently, there was negative value in lime stabilization because of necessary absorption of CO_2_ in the second step in hydration reaction. Therefore, this portion of GHG emissions reduction should be taken to the consideration of direct emission unit. Beside mentioned above, another similar CO_2_ capture is from lime synthesis occurred in lime production stage, this portion of carbon emission decrease was fitted into the list of carbon credit, meaning that a big piece of cake to be reckoned was potentially pointed out and put on an important position.

The reduction of 0.8092 tCO_2_e∙t^−1^ of sludge due to lime treatment is well-founded to obtain CERs (Certified Emission Reductions), which meets the principles and requirements of the CDM and is conducive to financial aid from developed countries to developing countries under the CDM frame^18^. By 2016, there was a capacity of approximately 350 tons sludge everyday in Qian’an city. Accordingly, if half of sludge production was treated by lime stabilization instead of landfill, considerable potential CERs of approximate 44, 340 tCO_2_e will be obtained annually. This portion of carbon credit can be incorporated together with that from the treatment of other kind of waste, then bound to a CERs package for sale.

In practice, most serious bottleneck is subsequent recycling of limed sludge. If it was incinerated, then lime pretreatment of sludge is unnecessary^12^. If it was bio-treated by aerobic compost or anaerobic digestion, the essential microbial groups had been inactivated by the heat released in lime stabilization and its strong alkalinity. Moreover, the organic matter reduction drastically was found in the process of lime addition to sludge^19^, in other word, limed sludge is not feasible to be bio-treated theoretically. It seems that only remaining way is suitable for limed sludge, which is amendment of limed sludge to acid soil for vegetation restoration. The abundant nutrients contained in sludge can be recycled; meanwhile, soil acidity is able to be neutralized, which is beneficial for reduction in heavy metal availability and mobility further. Consequently, the benefit of limed sludge amended soil to environmental safety is certainly increased.

## Materials and Methods

### Description of baseline scenario and its quantification of GHG emissions

About 65–70% of sludge was disposed by landfill in China^20^, so landfill is usually acceptable to become a baseline scenario in sludge relative investigation. This work investigated a sludge treatment system in Qian’an (a city in northeastern Hebei Province, China) where landfill accounts for 90% of sludge fate. Meanwhile, there is no environmental law to regulate and control the sludge treatment or disposal. Recovery facilities of landfill generated gas are not constructed widely in China. Therefore, sludge landfill without generated gas capture was defined as the baseline scenario in present work.

Sludge landfill produced total GHG emissions (PE_*TD,y*_) were quantified according to following formula:

1 $$P{E}_{TD,y}=P{E}_{elec,y}+P{E}_{fuel,y}+P{E}_{tran,y}+P{E}_{d,y}$$

PE_*elec,y*_: on-site emission from electricity use activity in landfill (tCO_2_e);

PE_*fuel,y*_: on-site emission from fuel use in landfill activity (tCO_2_e);

PE_*tran,y*_: leakage emission led by increased sludge transport (tCO_2_e);

PE_*d,y*_: direct emission from sludge landfill (tCO_2_e).

2 $$P{E}_{elec,y}=P{E}_{PJ,FF}\times CE{F}_{elec}$$

EG_*PJ,FF,y*_: electricity consumption from power grid triggered by sludge landfill, which was monitored by electricity meter (MWh);

CEF_*elec*_: electricity generation carbon emission factor related with sludge landfill (tCO_2_∙MWh^−1^), it was up to the basis of *Notification on Determining Baseline Emission Factors of China’s Grid*^21^. In China, there were seven different regional carbon emission factors including north China, northeast China, east China, northwest China, central China and south China. Here, it was defaulted to the grid average in China in 2012, which was given to 0.806.

3 $$P{E}_{fuel,on-site,y}={F}_{cons,y}\times NC{V}_{fuel}\times E{F}_{fuel}$$

F_*cons,y*_: amount of fuel consumption (l or kg);

NCV_*fuel*_: fuel’s net caloric value (MJ∙kg^−1^ or MJ∙l^−1^), it was taken as 42,652 KJ∙kg^−1^ here based on *Notification on Determining Baseline Emission Factors of China’s Grid*^21^;

EF_*fuel*_: CO_2_ emissions factor of fuel (tCO_2_∙MJ^−1^), it was selected to adopt as 72,600 kgCO_2_∙TJ^−1^ according to *Notification on Determining Baseline Emission Factors of China’s Grid*^21^.

4 $${{\rm{PE}}}_{\mathrm{tran}{,}y}=\sum _{i}^{n}{{\rm{NO}}}_{\mathrm{vehicles}{,}i{,}y}\times {{\rm{DT}}}_{i{,}y}\times {{\rm{VF}}}_{\mathrm{cons}{,}i}\times {{\rm{NCV}}}_{{fuel}}\times {D}_{{fuel}}\times {{\rm{EF}}}_{{fuel}}$$

*NO*_*vehicles,i,y*_: number of vehicles for sludge transport;

DT_*i,y*_: average additional distance travelled by vehicle (km);

VF_*cons,I*_: amount of vehicle fuel consumption (l∙km^−1^);

NCV_*fuel*_: fuel calorific value (MJ∙kg^−1^);

D_*fuel*_: fuel density (kg∙l^−1^);

EF_*fue*l_: emission factor of fuel (tCO_2_e∙MJ^−1^), it was set to 72,600 kg∙kJ^−1^ in term of *2006 IPCC Guidelines for National Greenhouse Gas Inventories*^22^.

5 $$P{E}_{d,y}=M{B}_{y}-M{D}_{reg,y}$$

MB_*y*_: emission of methane (CH_4_) resulted from sludge landfill (tCO_2_e);

MD_*reg,y*_: quantity of gas flaring or collection (tCO_2_e).

6 $$\begin{array}{rcl}{{\rm{MB}}}_{y} & = & {\rm{\phi }}\times (1-{f)\times \mathrm{GWP}}_{{{\rm{CH}}}_{4}}\times (1-\mathrm{OX})\\  &  & \times \frac{{\rm{16}}}{{\rm{12}}}\times {\rm{F}}\times {{\rm{DOC}}}_{f}\times {\rm{MCF}}\times \sum _{x=1}^{y}\sum _{i}{W}_{j,x}\\  &  & \times \,{{\rm{DOC}}}_{j}\times {e}^{-{k}_{j}\cdot (y-x)}\times (1-{e}^{-{k}_{j}})\end{array}$$

φ: model uncertainties correction factor, it was valued to be 0.9 in this case;

f: ratio of captured methane in manner of flare up, combustion or other manner, it was defaulted to 0 here;

GWP_CH4_: methane’s global warming potential (GWP) (tCO_2e_·t^−1^CH_4_), it was 25 here;

OX: oxidation factor, it was defaulted to 0 in this work;

F: fraction of methane in biogas, it was selected to be 0.5 here;

DOC_f_: fraction of degradable organic carbon in sludge, it was defaulted to 0.5 in this work;

MCF: methane correction factor, it was adopted to 1.0 here;

W: amount of not disposed sludge (t);

DOC_*j*_: ratio of degradable organic carbon, it was defaulted to 0.5 in this case;

K: ratio of sludge being rotten;

x: number of year at range of crediting period;

y: number of year for methane emission being accounted.

### Quantitative description of GHG emissions due to lime stabilization

Total GHG emissions (PE_*TLS,y*_) from lime stabilization is quantified according to following equation:

7 $${{\rm{PE}}}_{TLS,y}={{\rm{PE}}}_{elec,y}+{{\rm{PE}}}_{fuel,on-site,y}+{{\rm{PE}}}_{tran,y}+{{\rm{PE}}}_{c,y}+{{\rm{PE}}}_{lime,y}$$

PE_*elec,y*_: on-site emission attributed to electricity use activity (tCO_2_e);

PE_*fuel,y*_: on-site emission for fuel use activity (tCO_2_e);

PE_*tran,y*_: leakage emission triggered by increased sludge transport (tCO_2_e);

PE_c,y_: direct emission from sludge lime stabilization (tCO_2_e);

PE_*lime,y*_: indirect emission due to amount of lime input in stabilization reaction (tCO_2_e);

PE_*elec,y*_, PE_*fuel,y*_ and PE_*tran,y*_ were calculated in like manner as described in section 2.1.

8 $${{\rm{PE}}}_{c,y}={{\rm{PE}}}_{c,N2O,y}+{{\rm{PE}}}_{c,CH4,y}={{\rm{EF}}}_{c,N2O}\times {{\rm{GWP}}}_{N2O}\times {{\rm{M}}}_{compost,y}+{{\rm{BE}}}_{CH4,SWDS,y}\times {{\rm{S}}}_{a,y}$$

PE_*c,N2O,y*_: nitrous oxide (N_2_O) emission during lime stabilization reaction process (tCO_2_e);

PE_*c,CH4,y*_: methane emission during lime stabilization reaction process (tCO_2_e);

EF_*c,N2O*_: an emission factor for N_2_O (kgN_2_O·t^−1^), it was taken as 0.043 here^23^;

GWP_*N2O*_: global warming potential of N_2_O (tCO_2e_·t^−1^N_2_O), it was set to 310 in this work^24^;

BE_*CH4,SWDS,y*_: CH_4_ generation due to sludge landfill without capture facility (tCH_4_);

S_*a,y*_: in anaerobic condition, ratio of the decomposed sludge during landfill (%);

PE_*lime,y*_ was accounted for indirect emission due to lime addition activity.

9 $${{\rm{PE}}}_{\mathrm{lime},y}={{\rm{W}}}_{{\rm{lime}}}\times {{\rm{EF}}}_{{\rm{lime}}}$$

W_lime_: amount of lime input (t);

EF_lime_: an emission factor of lime synthesis or production (tCO_2_e·t^−1^), it was adopted as 0.4 here^25^
